# Genome-wide identification of the mango *CONSTANS* (*CO*) family and functional analysis of two *MiCOL9* genes in transgenic *Arabidopsis*

**DOI:** 10.3389/fpls.2022.1028987

**Published:** 2022-10-17

**Authors:** Yuan Liu, Cong Luo, Rongzhen Liang, Moying Lan, Haixia Yu, Yihang Guo, Shuquan Chen, Tingting Lu, Xiao Mo, Xinhua He

**Affiliations:** ^1^ State Key Laboratory for Conservation and Utilization of Subtropical Agro-Bioresources, National Demonstration Center for Experimental Plant Science Education, College of Agriculture, Guangxi University, Nanning, China; ^2^ Guangxi Key Laboratory for Agro-Environment and Agro-Product Safety, National Demonstration Center for Experimental Plant Science Education, College of Agriculture, Guangxi University, Nanning, China

**Keywords:** mango, *CONSTANS*, genome-wide analysis, expression pattern, function analysis

## Abstract

*CONSTANS*/*CONSTANS*-*like* (*CO*/*COL*) transcription factors play a vital role in the photoperiodic flowering pathway. However, the biological functions of *COL* genes in mango are unclear. In this study, we identified 31 *COL* genes from the ‘Jin Huang’ mango genome and divided them into three groups according to the specific gene structure and protein domain characteristics. These 31 *MiCOL* genes were heterogeneously distributed on 14 chromosomes. Expression pattern analysis showed that most *MiCOL* genes were mainly expressed in leaves and stems and during the floral induction period, followed by the floral differentiation period. The expression of *COL* genes was induced by drought and salt stress, but the expression patterns of different genes were different, which may suggest that *MiCOL* genes are involved in the abiotic stress response of mango. Under salt and drought conditions, two *MiCOL9* genes can improve the resistance of *Arabidopsis* by improving the scavenging ability of ROS and proline accumulation and reducing the MDA content. Additionally, overexpression of *MiCOL9* genes significantly inhibited flowering in transgenic *Arabidopsis*. This work provides an important foundation for understanding the biological roles of mango *COL* genes in plant growth, development and stress responses.

## Introduction

Many plants sense photoperiod information to predict impending environmental changes and precisely regulate flowering time under favorable conditions (Thomas and Vince-Prue, 1997). *CONSTANS* (*CO*) is a key gene in the photoperiod pathway and activates the downstream target gene *FLOWERING LOCUS T* (*FT*) in leaves. The FT protein is transferred from the phloem of the leaf to the apical meristem of the stem, forming a complex with *FLOWERING LOCUS D* (*FD*) and promoting the expression of the downstream flowering gene *APETALA1* (*AP1*), thereby inducing plant flowering (Kinmonth-Schultz et al., 2016; Putterill and Varkonyi-Gasic., 2016). The expression rhythm of the *CO* gene is regulated by the circadian clock, but the circadian clock does not directly act on the *CO* gene; rather, through circadian rhythm–regulating components such as *GIGANTIA* (*GI*), *CYCLING DOF FACTORS* (*CDF*) and the F-box protein *FLAVIN BINDING*, *KELCHREPEAT* (*FKF1*), transcriptional regulation is performed (Fowler et al., 1999; Huq et al., 2000; Imaizumi et al., 2005; Sawa et al., 2007; Fornara et al., 2009).

*CO* is a unique type of transcription factor (TF) in the plant kingdom (Putterill et al., 1995) that consists of two conserved domains defined by zinc finger TF domains: the B-box zinc finger region, located near the amino terminus, which is a key domain for protein–protein interactions, and the CCT domain, a region of 43 amino acids near the carboxy terminus that may mediate protein–DNA binding (Khanna et al., 2009).

Often, the second B-box mutates in the two B-box structures, and this rich variability may contribute to the diversity of CONSTANS-like (COL) protein functions (Song et al., 2012; Simon et al., 2015; Mulki and von, 2016). However, the nucleotide diversity of most genes is significantly reduced in the CCT domain, indicating that the CCT domain is highly conserved (Xiao et al., 2018). The CCT domain forms a trimeric CO/AtHAP3/AtHAP5 complex and binds to the CCAAT box in the eukaryotic promoter to regulate *Arabidopsis* flowering through FT expression (Wenkel et al., 2006). Thus, the strong conservation of the CCT domain is thought to be necessary for its role in controlling photoperiod flowering.

In total, 854 putative *COL* genes from 81 species are collected in the PlantTFDB (Jin et al., 2014). Sixteen kinds of *CO* family genes isolated from *Arabidopsis* are classified into three categories. Sixteen *OsCOL* genes have also been isolated from rice (*Oryza sativa*) (Griffiths et al., 2003) and divided into two groups: the genes in the first group contain two incomplete B-boxes, such as *OsCO3*, which inhibits flowering under short-day conditions, while the genes in the second group do not have a typical B-box domain but contain a CCT domain, such as *OsCO7*, which inhibits flowering under long-day conditions (Xue et al., 2008). In one study, 17 flowering genes of longan (*Dimocarpus longana Lour*.) were screened and divided into five categories (Zhai et al., 2016). In addition, 15 *PbCOL* genes have been identified in Chinese white pear (*Pyrus bretschneideri*) and divided into three categories (Wang et al., 2017). The *ClCOL* genes (*ClCOL*-*ClCOL11*) in *Chrysanthemum lavandulifolium* are divided into three categories. The class I genes (*ClCOL4*-*ClCOL5*) are highly transcribed under light conditions. The class II genes (*ClCOL*1-*ClCOL2*, *ClCOL10*) show increased expression in the dark and are rapidly inhibited by illumination. *ClCOL6*-*ClCOL9* and *ClCOL11* belong to class III and have high expression levels at the beginning of the dark period (Fu et al., 2015). In addition, 19 candidate homologous *GbCO* genes have been screened in *Ginkgo biloba* (Guan et al., 2016; Yan et al., 2017).

COL is a key gene in the photoperiod pathway, but the flowering of mango does not depend on the length of light (Luo et al., 2019). Therefore, what role does COL play in the flowering process of mango? *COL* genes have multiple copies and functions in many woody plants. It has been reported that the expression of *COL* genes in rice (Kim et al., 2008), a *Phalaenopsis hybrid* (Zhang et al., 2011a), barley (Campoli et al., 2012), sorghum (Yang et al., 2014), and red oak (Lind-Riehl et al., 2014) is related to the photoperiodic flowering regulation response. Although the COL gene has been isolated and cloned in many species and *MiCO*, *MiCOL1* and *MiCOL16* of mango have also been reported, the mango COL gene family has not been reported. Therefore, based on previous studies, this study obtained data on the *COL* gene family from the genome of ‘Jin Huang’ mango (data unpublished). The sequences were bioinformatically assessed, and the expression patterns were explored. The functions of the two *MiCOL9* genes were further analyzed. In total, we obtained 31 *COL* genes. The expression of these genes was similar in all tissues of mango and was regulated by stress. Overexpression of the two *MiCOL9* genes significantly inhibited flowering and improved the resistance of transgenic *Arabidopsis* to salt and drought. This study lays a foundation for the study of *COL* gene function in mango.

## Materials and methods

### Plant materials

In this study, five-year-old mango trees were grown in the fruit tree specimen garden of Guangxi University, Nanning, Guangxi, China (latitude 108°22’N, longitude 22°48’E; elevation 79.5 m). The tender leaves, mature leaves, mature stems and flowers for tissue expression analysis were collected on March 11, 2017, and leaves for temporal expression analysis were collected once per month from November 1, 2016, to March 11, 2017. One-year-old mango trees were treated with 1 L 300 mM NaCl solution for salt treatment, and 1 L 30% polyethylene glycol 6000 (PEG6000) for drought treatment; water treatment was used as a control. The leaves were collected at 0 h, 6 h, 12 h, 24 h, 48 h and 72 h after treatment. All samples were frozen in liquid nitrogen immediately and kept at -80°C.

### Identification of *CO/COL* genes in mango

To identify the *MiCOL* genes in the mango genome (data unpublished), multiple database searches were performed. The protein sequences of *COL* in *Arabidopsis* were downloaded from The *Arabidopsis* Information Resource (TAIR, http://www.arabidopsis.org/) and were used as a query to conduct a local blast search against mango genome databases. The predicted proteins from mango genome databases were searched by HMMER v3 (Eddy, 1995) using a hidden Markov model (HMM) file that corresponded to the B-box zinc finger domain (PF00643) and CCT motif (PF06203) downloaded from the Pfam database (http://pfam.xfam.org/) as a query under an E-value< 1.0 (El-Gebali et al., 2019). Then, the existence of B-Box and CCT domains for all identified sequences was confirmed by a conserved domain search (CD-Search) of the National Center for Biotechnology Information (https://blast.ncbi.nlm.nih.gov/). Incomplete sequences and unqualified conserved domains were removed from the results. Finally, the filtered protein sequences were regarded as *CO* TFs of *Mangifera indica* L. (*MiCO*s) for subsequent evaluation.

### Phylogenetic analysis

Multiple sequence alignment of AtCO and MiCO proteins was performed using ClustalX 2.1 with the default settings (Thompson et al., 1994). An unrooted phylogenetic tree was constructed with MEGA software (version 6.06) using the neighbor-joining method with 1000 bootstrap replicates (Tamura et al., 2013). iTOL (Letunic and Bork, 2007) (https://itol.embl.de/) and EvolView (He et al., 2016) (https://evolgenius.info/evolview-v2/) were used for phylogenetic tree visualization and annotations.

### Analysis of structures and conserved motifs of proteins

The conserved motifs in MiCO proteins were analyzed by Multiple Em for Motif Elicitation (MEME; version 5.41, https://meme-suite.org/meme/tools/meme) (Bailey et al., 2015). The maximum number of motifs was set to 10, and all other parameters were the default ones. Motif analysis and gene structure visualization were performed *via* TBtools (version 1.098728) (Chen et al., 2020).

### Promoter cis-element analysis

The 3000 bp upstream promoter sequences of *MiCOL* genes were collected. The promoter sequences were uploaded to the PlantCARE database (Lescot et al., 2002) (http://bioinformatics.psb.ugent.be/webtools/plantcare/html/), and the cis-elements were subsequently screened manually. The identities and locations of the cis-elements were visualized *via* TBtools (Chen et al., 2020).

### Chromosomal distribution and gene duplication

The lengths of chromosomes and the locations of *MiCOL* genes in mango were identified according to the mango genome database, and a homology analysis was performed among *MiCOL* family members. To deduce the evolutionary relationships of CO genes among different species and the *MiCOL* genes among different mango varieties, syntenic analysis was performed for three species (mango, grape and *Populus trichocarpa*) (Wang et al., 2019; Li et al., 2020a) and three mango varieties (‘JinHuang’, ‘Alfonso’ and ‘Tommy Atkins’) (Sherman et al., 2015; Wang et al., 2020) (https://phytozome-next.jgi.doe.gov/;https://www.ncbi.nlm.nih.gov/genome) with MCScanX software (Wang et al., 2012). Collinearity analysis was performed, and chromosomal location diagrams were generated in a globe plot using the program TBtools (version 1.098728) (Chen et al., 2020).

### qRT–PCR analysis

Total RNA was extracted from the above samples using an RNAprep Pure Kit (for polysaccharide- and polyphenolic-rich plants, DP441) (Tiangen, Beijing, China), and first-strand cDNA was synthesized with a PrimeScript Reverse Transcriptase Kit (Takara; Dalian, China). The sequences of the internal control genes *MiActin1* and *MiCOL* amplified by gene-specific primers are shown in **Supplementary Table S1** (Luo et al., 2013). Quantitative real-time PCR (qRT–PCR) analysis was performed with an ABI 7500 Real-Time PCR System (Applied Biosystems, Foster City, CA, USA) using SYBR Premix Ex Taq II (Takara, Dalian, China). The expression data were normalized according to the 2^−ΔΔCt^ method (Livak and Schmittgen, 2001) and are shown in a heatmap that was created using TBtools (version 1.098728) (Chen et al., 2020). Three technical repetitions were assessed per sample.

### Subcellular localization and transcriptional activity analysis

The *MiCOL9A* and *MiCOL9B* coding sequences without a stop codon were inserted into the pBI121 vector, and then *MiCOL9A*, *MiCOL9B* and GFP were used to form the *35S*::*MiCOL9A*-*GFP* and *35S*::*MiCOL9B*-*GFP* fusion expression vectors. The correct constructs were subsequently transformed into onion epidermal cells by *Agrobacterium tumefaciens* (GV3101), and the fluorescent cells were observed *via* laser confocal microscopy (TCS-SP8MP; Leica, Germany). The empty GFP vector was used as a negative control, and 4’,6-diamidino-2-phenylindole (DAPI) was used to visualize the nucleus.

To analyze the transcriptional activity of the *MiCOL9A* and *MiCOL9B* genes, and the fulllength CDS and middle region (MR) deletion of *MiCOL9A* and *MiCOL9B* were amplified *via* PCR and inserted into the pGBKT7 expression vector, to generate the *BD-MiCOL9A*, *BD-MiCOL9B*, *BD-MiCOL9A-ΔMR* and *BD-MiCOL9B-ΔMR* constructs (Clontech, Dalian, China), and the constructs were subsequently transferred into Y2H Gold yeast cells. The transformed products were cultured and detected on SD/-Trp, SD/-Trp/X-α-gal and SD/-Trp/X-α-gal/AbA media. The empty pGBKT7 vector was used as a control.

### *Arabidopsis* transformation

The coding sequences of the MiCOL9A and MiCOL9B genes were inserted respectively into the pBI121 vector *via* the CaMV 35S promoter control. *Agrobacterium tumefaciens* (EHA105) containing positive recombinant plasmids was transformed into *Arabidopsis* (Col-0) plants with the floral-dip method (Clough and Bent, 1998). For the third-generation (T3) homozygous positive transformants, RNA was extracted for semiquantitative RT–PCR (SqRT-PCR)-based analysis before flowering (Wang et al., 2021), and the flowering-related genes *AtFT*, *AtSOC1* and *AtFLC* were used for qRT−PCR analysis (**Table S1**). *AtActin2* was used as a reference control. The flowering time and rosette leaves were measured when blooming. The number of samples of each line was at least 15.

### Transgenic and WT lines under stress treatment

The *MiCOL9A* and *MiCOL9B* transgenic lines and wild-type (WT) seeds were sown onto 1/2 MS media without antibiotics and vernalized for 2 days. After 3 days of growth at 22°C, the transgenic lines and WT seedlings were transformed onto 1/2 MS media supplemented with NaCl (0, 100, 150 and 200 mM) or mannitol (0, 300, 400 and 500 mM) and then grown under LD conditions at 22°C. Approximately 7 days later, the length of primary roots and fresh weight were measured. Three biological repetitions were assessed per sample.

Seven-day-old T3 homozygous transgenic lines and WT seedlings were cultivated in soil for one week. For drought treatment, the WT and transgenic plants were denied water for two weeks and then rewatered for 3 days, and the survival rate was measured. For salt treatment, approximately 3-week-old seedlings were treated with 300 mM NaCl solution for 6 days, and the survival rate was measured. The transgenic lines and WT seedlings were treated with water as a control, and three biological repetitions were assessed per sample.

To determine the expression pattern of stress-related genes in transgenic plants under salt and drought stress conditions, the total RNA of 15-day-old transgenic lines and WT plants was extracted. The specific primers for the salt-related genes *AtCOR47*, *AtNHX1*, and *AtRD29A* and the drought-related genes *AtKIN1*, *AtNCED3*, and *AtRD29B* were used for qRT−PCR (**Table S1**). *AtActin2* was used as a reference control. Three biological repetitions were assessed per sample.

### Histochemical staining and determination of physiological indexes

To explore the accumulation of reactive oxygen species (ROS) and cell death of transgenic and WT lines under salt and drought stress, histochemical staining was used. Three-week-old transgenic and WT plants were treated with water (as a control), 150 mM NaCl and 200 mM mannitol for 3 h, and the leaves were cut for staining and placed into 3,3′-diaminobenzidine (DAB) (1.0 mg/mL) for 8 h, nitrotetrazolium blue chloride (NBT) (0.5 mg/mL) for 3 h and Evans blue (2.5%, w/v) for 15 min (Kim et al., 2003; Zhang et al., 2011b; Li et al., 2020b). Then, the strain leaves were decolorized in 80% ethanol.

For determination of physiological indexes, the leaves of 3-week-old transgenic and WT plants were treated with water (as a control), and 150 mM NaCl and 200 mM mannitol for 3 days were cut. The MDA, SOD, POD, and proline contents were measured using reagent kits (Solarbio Science and Technology, Beijing, China), and the H_2_O_2_ and ${\text{O}}_{2}^{-}$ contents were measured using reagent kits (Grace Biotechnology, Jiangsu, China).

### Interaction protein validation

The plasmids of interacting proteins which screened by Y2H assay were extracted, and transferred into Y187 yeast cells. The pGBKT7-bait in Y2H Gold yeast cells and candidate prey in Y187 yeast cells were mixed into 2×YPDA liquid medium at 30°C and 200 rpm for 20-24 h. The mixture were coated on SD/-Trp/-Leu (DDO) and SD/-Trp/-Leu/-His/-Ade/X-α-gal/AbA (QDO/X/A) mediums for 3-5 days. The pGBKT7-vector (BD-T7) was used to detect the self-activation of candidate proteins.

To further verify the role of the *MiCOL9A* and *MiCOL9B* genes in flowering and stress, the bimolecular fluorescence complementation (BiFC) assay was performed. Three flowering-related genes, vascular plant zinc-finger 1A (VOZ1A), VOZ1B, and VOZ1C, and six stress-related genes, zinc-finger protein 4 (ZFP4), RING zinc finger protein 34 (RZFP34), MYB30-interacting E3 ligase 1 (MIEL1), NAC7, ubiquitin-conjugating enzyme E2 (UBE2C) and protein phosphatase 2C39A (PP2C39A), were selected. The full-length CDS was inserted into the pSPYCE vector, and the full-length CDS of the *MiCOL9A* and *MiCOL9B* genes was inserted into the pSPYNE vector. All fusion constructs were transformed into *A. tumefaciens* (strain GV3101), and mixed bacterial fluid was transformed into *Nicotiana benthamiana* leaves. Two days later, the fluorescence signal was detected by laser confocal microscopy (TCS-SP8MP; Leica, Germany). DAPI was used to visualize the nucleus.

## Results

### Genome-wide identification of *CO* genes in mango

In total, 31 putative *CO* genes were identified from the mango genome (unpublished) (Xia et al., 2022; Zhu et al., 2022) using sequences of *Arabidopsis CO* genes as a reference (**Table 1**). The conserved domains of the target sequences were evaluated by bioinformatic analysis, and all putative genes were subsequently verified with HMMER and SMART (Simple Modular Architecture Research Tool) online software. The sequence lengths of *CO* genes in mango ranged from 492 bp to 1536 bp, the protein lengths ranged from 163 to 511 amino acids, the molecular weights (MWs) ranged from 18.11 to 56.63 kDa, and the isoelectric points of the predicted proteins ranged from 3.93 to 8.85. Prediction of subcellular localization suggested that all putative proteins might be targeted to the nucleus.

**Table 1:** Information concerning *MiCO* genes in mango.

Proposed gene name	GenBank ID	Chr	Orientation	CDS length (bp)	Protein length (aa)	Molecular weight (KDa)	PI	Predicted subcellular localization
MiCO	ON375428	12	–	969	322	35.28	5.83	Nucleus
MiCOL1A	ON375429	1	+	852	283	32.34	5.27	Nucleus
MiCOL1B	ON375430	17	+	822	273	31.01	4.89	Nucleus
MiCOL2A	ON375431	8	–	1119	372	41.22	5.31	Nucleus
MiCOL2B	ON375432	13	–	1110	369	41.08	6.66	Nucleus
MiCOL2C	ON375433	15	–	1023	340	38.66	5.15	Nucleus
MiCOL3A	ON375434	2	+	723	240	27.43	8.60	Nucleus
MiCOL3B	ON375435	8	+	720	239	27.31	7.34	Nucleus
MiCOL4A	ON375436	9	+	672	223	24.65	4.12	Nucleus
MiCOL4B	ON375437	16	–	492	163	18.11	4.40	Nucleus
MiCOL5A	ON375438	16	+	1068	355	39.51	6.68	Nucleus
MiCOL5B	ON375439	12	+	1083	360	39.33	6.41	Nucleus
MiCOL6	ON375440	2	–	681	226	26.89	4.55	Nucleus
MiCOL7A	ON375441	15	–	1056	351	39.88	8.07	Nucleus
MiCOL7B	ON375442	9	–	1017	338	38.59	8.85	Nucleus
MiCOL8	ON375443	7	–	591	196	23.46	4.63	Nucleus
MiCOL9A	ON375444	6	+	1257	418	44.96	5.16	Nucleus
MiCOL9B	ON375445	9	+	1260	419	45.63	4.70	Nucleus
MiCOL10	ON375446	4	+	1536	511	56.63	5.16	Nucleus
MiCOL11	ON375447	17	–	543	180	19.77	3.93	Nucleus
MiCOL12	ON375448	9	–	1272	423	46.55	4.62	Nucleus
MiCOL13A	ON375449	9	+	1128	375	42.56	6.82	Nucleus
MiCOL13B	ON375450	20	+	1158	385	43.66	6.55	Nucleus
MiCOL14A	ON375451	9	+	1176	391	44.14	4.81	Nucleus
MiCOL14B	ON375452	16	+	1134	377	41.42	4.48	Nucleus
MiCOL15A	ON375453	4	–	1470	489	54.29	5.86	Nucleus
MiCOL15B	ON375454	20	+	1434	477	53.05	7.23	Nucleus
MiCOL16A	ON375455	16	+	1272	423	47.06	5.57	Nucleus
MiCOL16B	ON375456	19	–	1254	417	46.71	7.06	Nucleus
MiCOL16C	ON375457	4	+	1371	456	51.03	7.24	Nucleus
MiCOL17	ON375458	3	+	1062	353	40.13	6.26	Nucleus

### Phylogenetic analysis of the CO proteins

**Figure 1:**
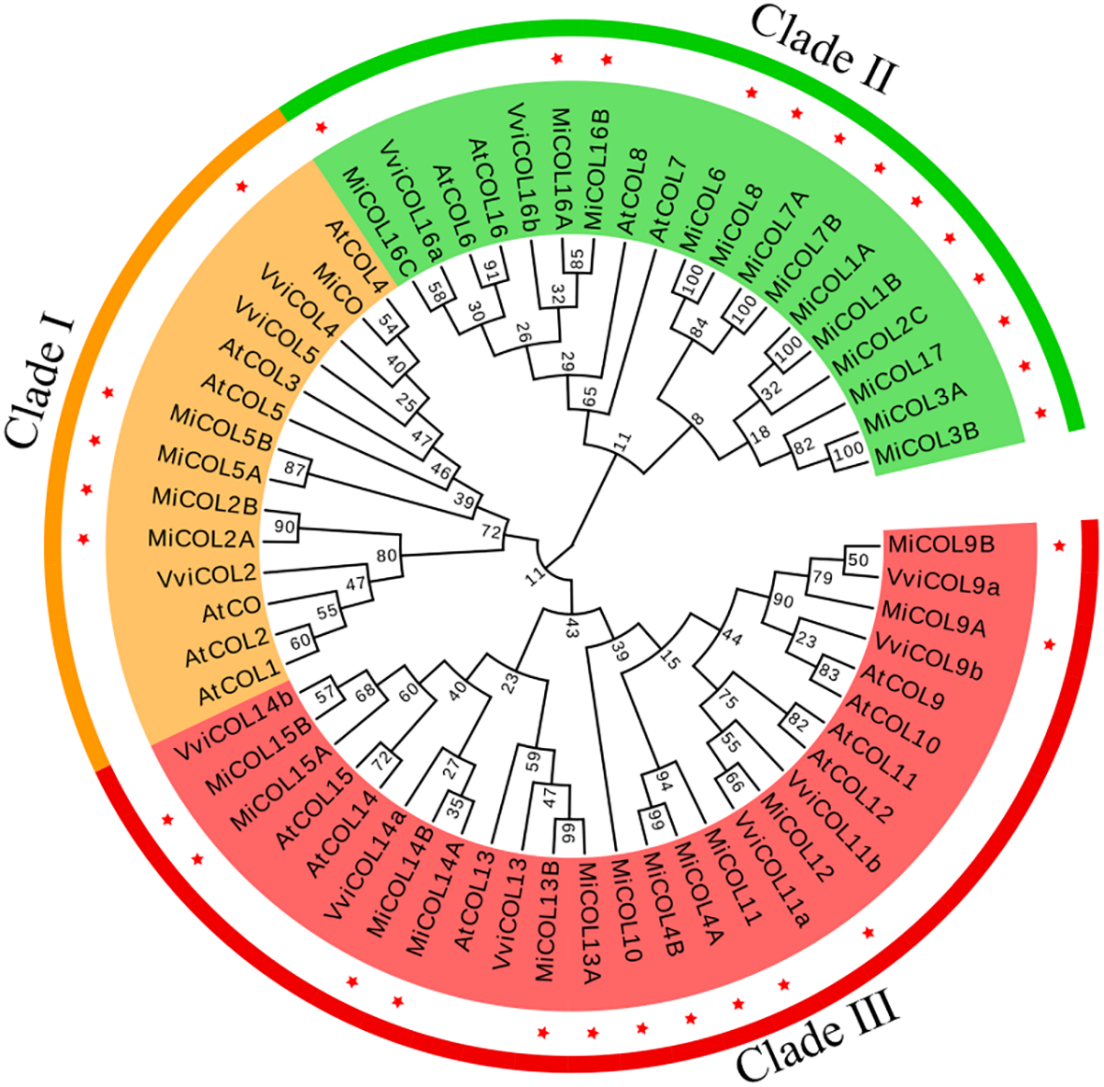
Phylogenetic tree of COL proteins in mango and *Arabidopsis*. Clades I, II and III are marked in yellow, green and red, respectively. Red stars represent mango family genes. The gene IDs of the COs/COLs in *Arabidopsis* are listed in **Supplementary Table S2**.

To analyze the evolutionary relationships of the COL proteins, a total of 60 COL proteins, including 17 from *Arabidopsis*, 12 from *Vitis vinifera* and 31 from mango, were used to construct a phylogenetic tree. As shown in **Figure 1**, the MiCOL proteins were resolved into three clades that mostly corresponded to their different structures. There were five MiCOL proteins in clade I that contained BBX1, BBX2, VP motifs and CCT domains and clustered with the AtCO and AtCOL1-5 proteins in group I of *Arabidopsis*. Thirteen MiCOL proteins that clustered with the proteins in group II of *Arabidopsis* were divided into clade II. There were two types in clade II: one type contained BBX1 and CCT domains and included MiCOL16A, MiCOL16B and MiCOL16C, and the other type contained only one CCT domain and included ten proteins. Thirteen MiCOL proteins were divided into clade III, which clustered with the proteins in group III of *Arabidopsis*. There were also two types in clade III: one type contained BBX1, DZF (diverged zinc finger) and CCT domains (MiCOL9A-MiCOL15B, except MiCOL11), and the other type contained only a BBX1 domain (MiCOL4A, MiCOL4B and MiCOL11) (**Figure 1**). Overall, this result indicated that the phylogeny of COL proteins in mango was similar to that in *Arabidopsis*.

### Gene structures and motif analysis

**Figure 2:**
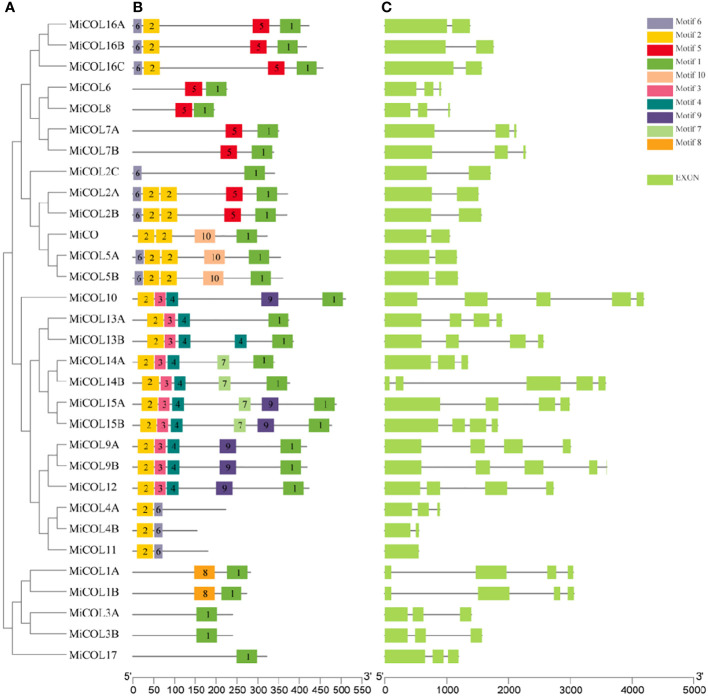
Phylogenetic, conserved motif, intron and exon structure analysis of the MiCOL genes in mango. **(A)** Phylogenetic analysis. **(B)** Conserved motif analysis. Different patterns are represented by different colors. **(C)** Gene structures. Exons and introns are represented by green rectangles and black lines, respectively.

To analyze the structure and conservation of the mango *CO* gene family, the conserved protein motifs and exon–intron structures were analyzed with the MEME and TBtools software programs inside (**Figure 2A**). Ten conserved motifs were identified. As shown in **Figure 2B**, motif 2, motif 1 and motif 3/4 corresponded to the B-box, CCT and DZF domains. Interestingly, motif 4 was present only in clade III and was accompanied by motif 3 and motif 1 in almost all CO genes except *MiCOL*4A, *MiCOL*4B and *MiCOL*11. Among these 31 *CO* genes, the genomic structures were quite different (**Figure 2C**). For intron number, 3 MiCOL genes contained 4 introns, 8 contained 3 introns, 9 contained 2 introns, 10 contained only 1 intron, and the *MiCOL*11 gene had no introns. The different numbers of exons/introns reflect the diversity of *CO* genes during evolution.

### Analysis of cis-acting regulatory elements (CAREs) in *MiCO* genes

**Figure 3:**
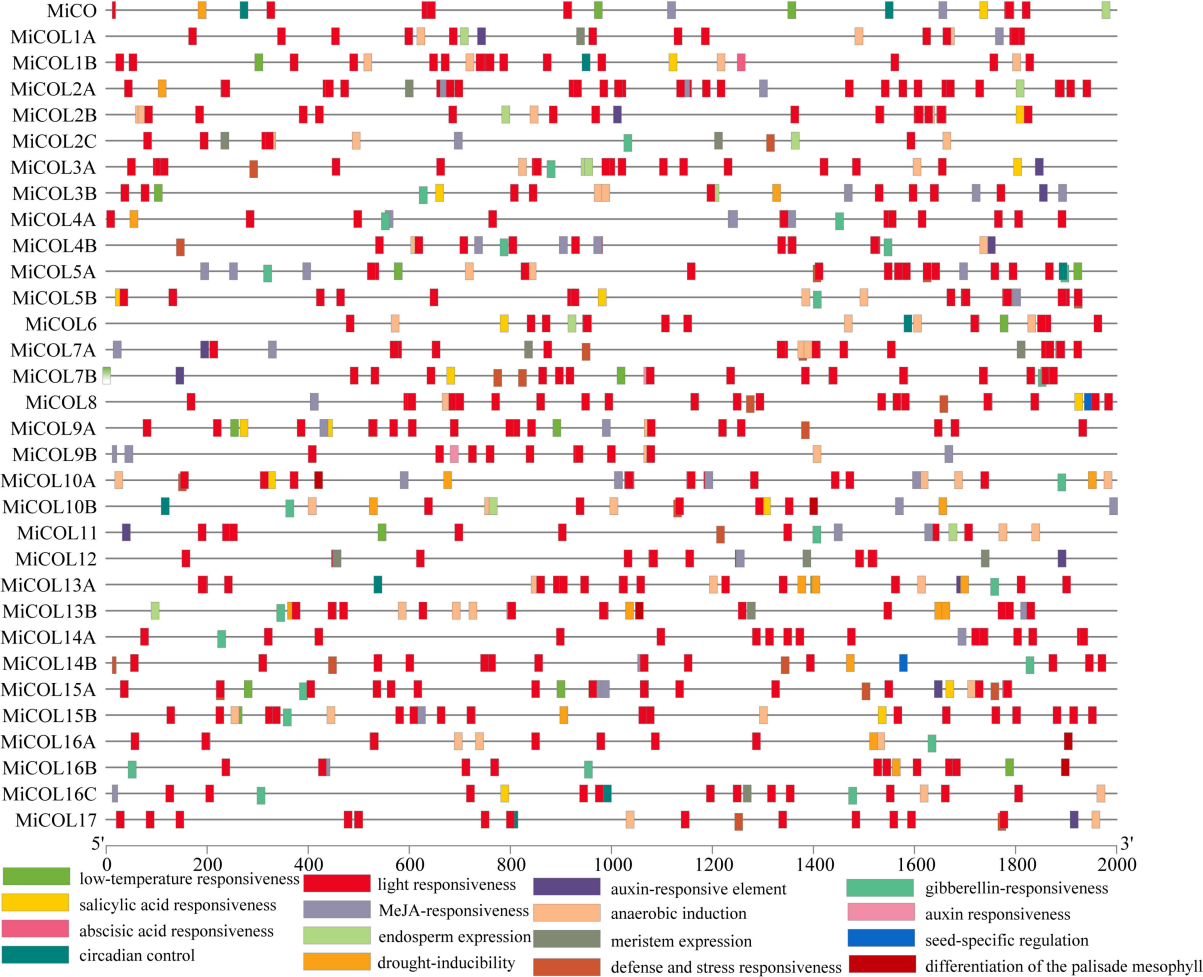
Analysis of cis-elements within the promoters of *MiCOL* gene family members.

To explore the potential regulatory mechanism of *MiCO* genes, cis-elements in the 2000 bp promoter region were analyzed *via* the PlantCARE database (**Figure 3**). Light-, circadian control-, hormone- and stress-responsive elements were widely distributed in the MiCO promoters. The results showed that a total of 16 unique CAREs were identified in the *MiCO* gene family, and the light responsiveness element was the most frequent. In addition, the *MiCO* genes were induced by many hormones, including MeJA-responsive, abscisic acid-responsive, gibberellin-responsive, salicylic acid-responsive and auxin-responsive elements. Most *MiCO* promoter regions contain a variety of stress-responsive elements, including those for drought inducibility and low-temperature responsiveness. The results suggested that *MiCO* gene family members mainly participate in photoperiod, circadian, hormone and stress pathways, indicating the diversity of *MiCO* functions.

### Chromosomal distribution and synteny analysis among *MiCO* genes

**Figure 4:**
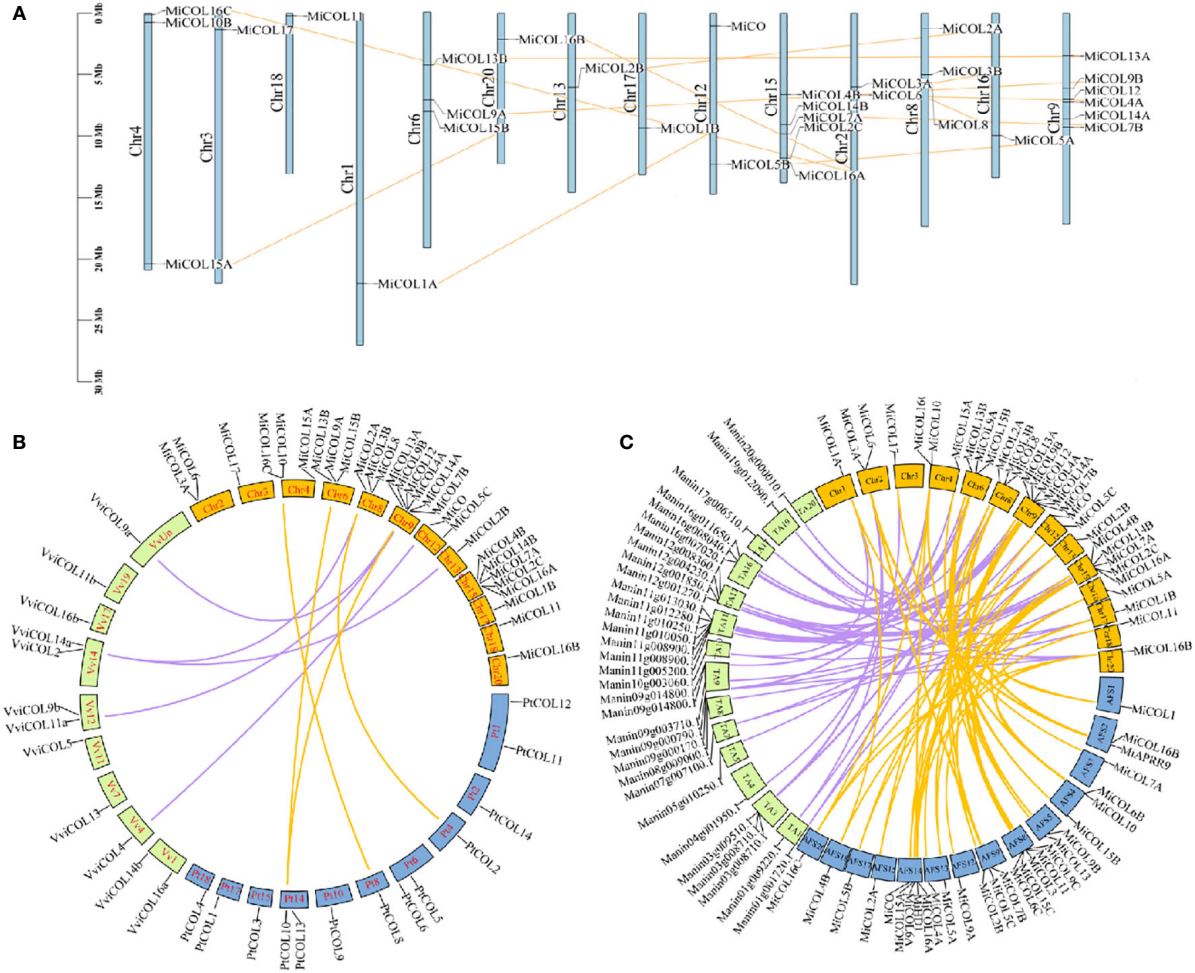
Chromosomal localization and synteny of *MiCOL*s. **(A)** The chromosome numbers are indicated at the top of each scaffold. The chromosome size is shown by the vertical scale. **(B)** Syntenic relationships of mango, *Vitis vinifera* and *Populus trichocarpa* COL genes. The colored lines represent the syntenic regions between mango and *Vitis vinifera* (purple) and between mango and *Populus trichocarpa* (yellow). **(C)** Syntenic relationships of *MiCOL* genes in different mango varieties. The colored lines represent the syntenic regions between ‘JinHuang’ and ‘Tommy Atkins’ (purple) and between ‘JinHuang’ and ‘Alfonso’ (yellow). The graph was generated *via* Circos.

To analyze the positions of *CO* gene family members in the mango genome, chromosomal mapping was performed, and gene duplication events were analyzed. The results suggested that 31 *MiCO* genes were heterogeneously distributed on 14 of the 20 chromosomes. Among these genes, there were six genes distributed on chromosome 9, which had the densest distribution, followed by chromosome 15, which had five genes. Chromosomes 4, 6, and 8 all had three genes each; chromosomes 2 and 12 both had two genes each; and the other seven chromosomes had only one gene each. Twelve pairs of repeat events were present in the *MiCO* gene family, and no tandem repeats were observed (**Figure 4A**).

To understand the origins and evolutionary relationships of the *COL*s, we further performed synteny analysis to compare the mango genome with the grape and *Populus trichocarpa* genomes. Five orthologous gene pairs were identified between mango and grape, and four pairs were identified between mango and *Populus trichocarpa* (**Figure 4B**). In addition, we also analyzed the synteny of the three mango varieties ‘JinHuang’, ‘Alfonso’ and ‘Tommy Atkins’, and the results showed that many orthologous gene pairs were identified (**Figure 4C**). These results indicated that these genes that were linked together might have descended from a common evolutionary ancestor.

### Expression profiles of *MiCO* genes in different tissues and over time

**Figure 5:**
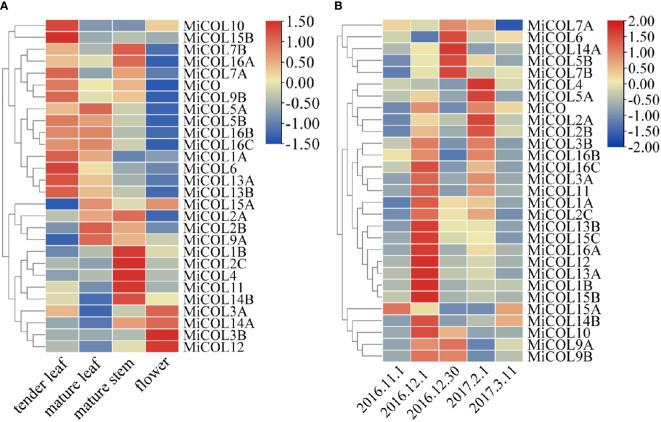
Tissue and temporal expression patterns of *MiCOL* genes in mango. **(A)** Expression levels in different tissues. **(B)** Expression levels in different growth periods. Vegetative growth period: November 1, 2016, to December 1, 2016. Floral induction period: December 1, 2016, to December 30, 2016. Floral differentiation period: December 30, 2016, to February 1, 2017. Inflorescence elongation and flowering period: February 1, 2017, to March 11, 2017. The color gradient (red/yellow/blue) indicates the gene expression level (from high to low).

To explore the expression patterns of *MiCO* genes in different tissues and at different times, qRT–PCR was performed. The results showed that *MiCO* genes were expressed in all tested tissues (**Figure 5A**). Most of the genes were mainly expressed in tender leaves, mature leaves and mature stems, which suggested that these genes may play important roles in the vegetative growth of mango. Some *MiCO* genes, such as *MiCOL3A*, *MiCOL3B*, *MiCOL12* and *MiCOL14A*, were mainly expressed in flowers and had low expression in other tissues, which indicates that these genes may play important roles in mango flowering development.

The results of temporal expression analysis suggested that all genes were expressed in the mango leaves at all tested periods (**Figure 5B**). The expression of the majority of genes increased during the vegetative growth period; the expression of the *MiCOL5B*, *MiCOL6*, *MiCOL7B*, *MiCOL9A*, *MiCOL9B* and *MiCOL14A* genes increased during the floral induction period; and the expression of the *MiCOL2A*, *MiCOL2B*, *MiCOL4* and *MiCOL5A* genes increased during the floral differentiation period. These results suggest that the *MiCO* gene family members mainly play important roles in mango vegetative growth and play relatively less-important roles in the flowering stage.

### Expression profiles of *MiCO* genes under abiotic stress

**Figure 6:**
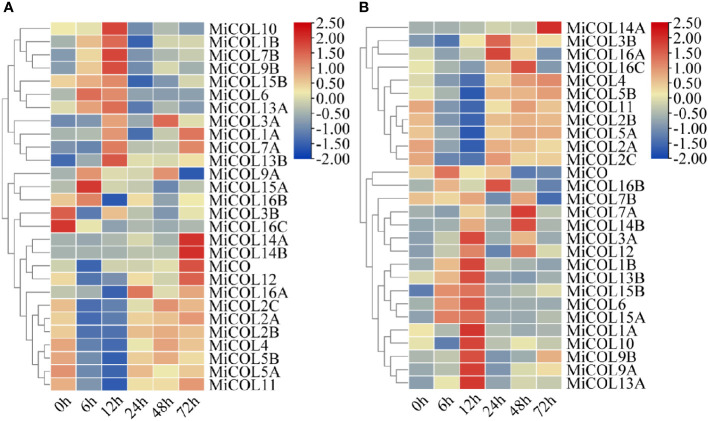
Expression patterns of *MiCOL* genes after different stress treatments. **(A)** Under 300 mM NaCl. **(B)** Under 30% PEG 6000 for drought simulation. The color gradient (red/yellow/blue) indicates the gene expression level (from high to low).

To determine whether the *MiCO* family genes are involved in the abiotic stress response, we analyzed the expression patterns of *MiCO* family genes in mango leaves under 300 mM NaCl and 30% polyethylene glycol 6000 (PEG 6000)-simulated drought conditions. The results indicated that the *MiCO* family genes had various expression patterns under different stress conditions. Under salt conditions, twenty-four genes showed no significant changes after salt treatment. The *MiCOL3B* and *MiCOL16C* genes were significantly downregulated, and the *MiCO*, *MiCO12*, *MiCOL14A* and *MiCOL14B* genes were significantly upregulated after salt treatment, and the *MiCOL9B* gene have the higher expression level after treatment 12 h (**Figure 6A**). This result indicates that these four genes may play important roles in the salt stress response. The *MiCOL14A* gene was significantly upregulated, and the *MiCO*, *MiCOL7B* and *MiCOL16B* genes were significantly downregulated after drought treatment, the *MiCOL9A* and *MiCOL9B* genes have the higher expression level after treatment 12 h (**Figure 6B**). This result indicates that the *MiCOL14A* gene may play a key role in the drought stress response.

In summary, among these genes, the *MiCOL9A* and *MiCOL9B* genes were mainly expressed during the floral induction period and had high expression levels after treatment, which suggests that these two genes may play indispensable roles in flowering regulation and the abiotic stress response. Thus, these two genes were selected for further functional studies.

### Subcellular and transcriptional activity analysis

**Figure 7:**
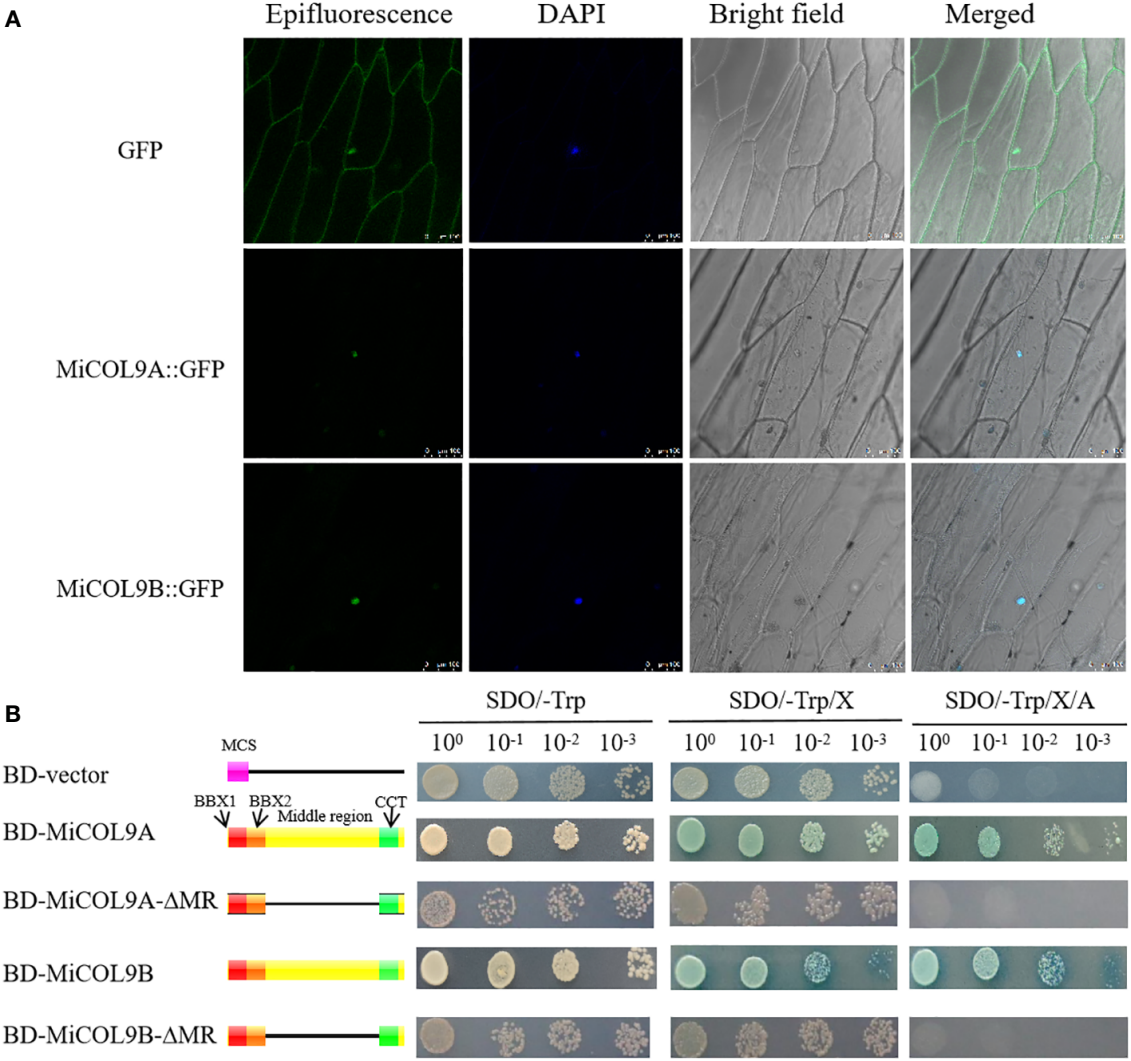
**(A)** Subcellular localization. Scale bars=100 µm. **(B)** Transcriptional activation activity. The left diagram shows various constructs. MCS, multiple cloning sites; BD, GAL4-DNA binding domain; BBX1, B-box 1; BBX2, B-box 2; CCT, CO, CO-like, TOC1; MR, middle region.

To evaluate the subcellular localization of the *MiCOL9A* and *MiCOL9B* genes, the *35S::MiCOL9A-GFP* and *35S::MiCOL9B-GFP* vectors were transformed into onion epidermal cells. The results showed that 35S:GFP localized to the nucleus and cytomembrane, while both *35S::MiCOL9A-GFP* and *35S::MiCOL9B-GFP* localized only to the nucleus (**Figure 7A**).

For transcriptional activity analysis, yeast cells, including empty pGBKT7 (BD-vector), *BD-MiCOL9A*, *BD-MiCOL9B*, *BD-MiCOL9A-ΔMR* and *BD-MiCOL9B-ΔMR* vectors, were transferred onto three selective media. Three days later, the BD-vector, *BD-MiCOL9A-ΔMR* and *BD-MiCOL9B-ΔMR* could not grow on SD/-Trp/X-α-gal/AbA medium, while *BD-MiCOL9A* and *BD-MiCOL9B* vectors grew on three medias, and turned blue on SD/-Trp/X-α-gal and SD/-Trp/X-α-gal/AbA media (**Figure 7B**). These results suggest that the *MiCOL9A* and *MiCOL9B* genes have transcriptional activation activity through their MR domain in yeast.

### Overexpression of *MiCOL9A* and *MiCOL9B* inhibits flowering in *Arabidopsis*

**Figure 8:**
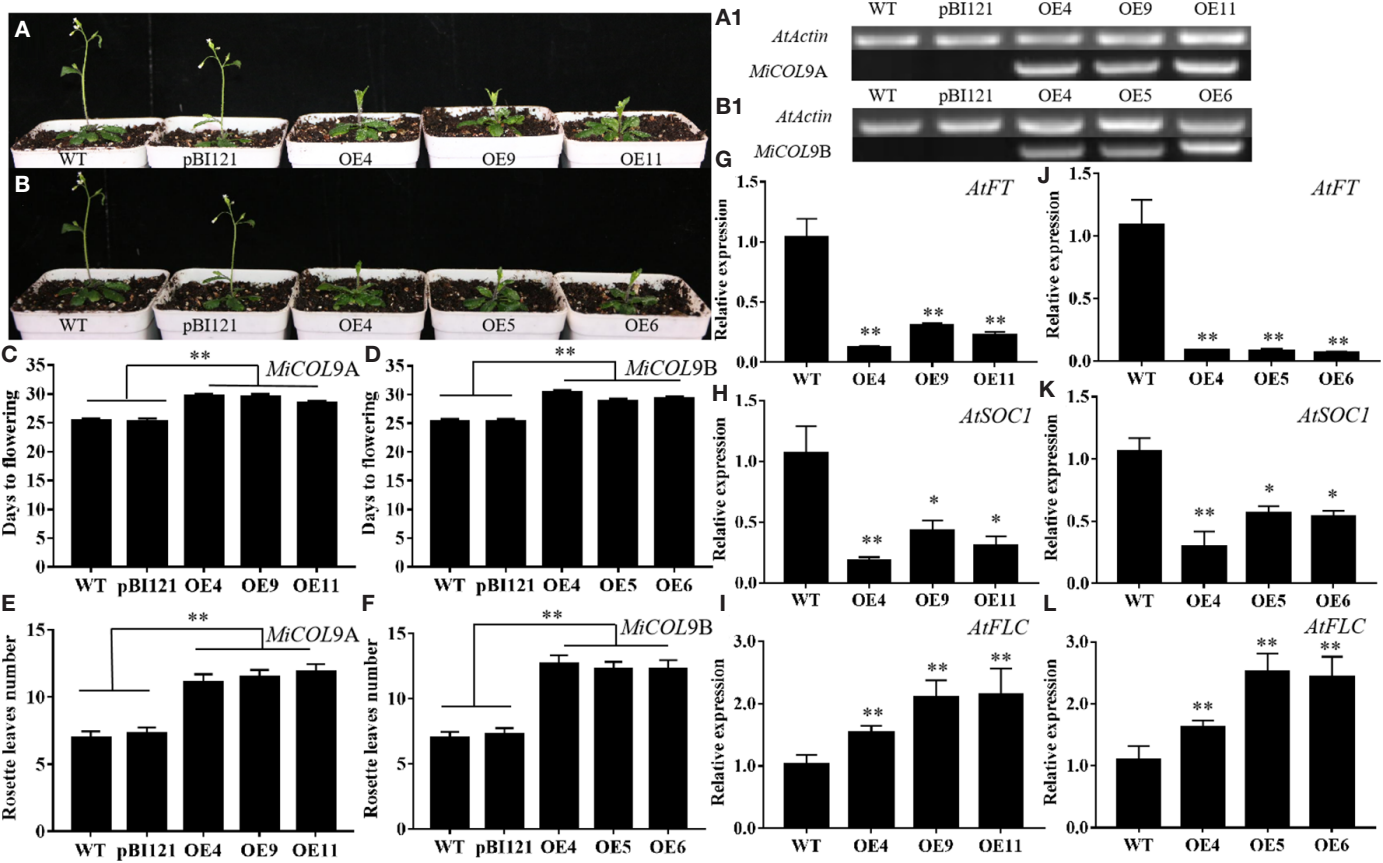
Ectopic expression of *MiCOL9A* and *MiCOL9B* delays flowering under LD conditions. **(A, B)** Phenotype of transgenic *Arabidopsis* plants under LD conditions. **(A1, B1)** sqRT-PCR of *MiCOL9A* and *MiCOL9B* in the WT, pBI121 and transgenic plants. **(C, D)** Flowering times. **(E, F)** Rosette leaf numbers. **(G-I)** Expression levels of *AtFT*, *AtSOC1* and *AtFLC* in *MiCOL9A* **(G-I)** and *MiCOL9B* **(J-L)** transgenic lines. Significant differences were assessed at *p*< 0.05 (*) and *p*< 0.01 (**) levels by Student’s *t* tests.

To determine the influence of the *MiCOL* genes on flowering time, three *MiCOL9A* and *MiCOL9B* 3rd generation transgenic lines were selected. The sqRT-PCR results showed that *MiCOL9A* and *MiCOL9B* were expressed only in transgenic lines (**Figures 8A1, B1**). Under LD conditions, the flowering time of *MiCOL9A*- and *MiCOL9B*-transgenic *Arabidopsis* was later than that of WT and empty vector-transformed (pBI121) plants (**Figures 8A-D**), and the number of rosette leaves of the transgenic line was significantly greater than that of the WT and pBI121 lines (**Figures 8E, F**). To further dissect the molecular mechanism of the *MiCOL9A* and *MiCOL9B* genes in flowering, we performed qRT−PCR of flowering-related genes. The results showed that the expression levels of two flowering-promoting genes, *AtFT* and *AtSOC1*, were significantly repressed, and the expression of the flowering inhibition gene *AtFLC* was significantly improved (**Figures 8G-L** **)**. These results indicated that overexpression of *MiCOL9A* and *MiCOL9B* leads to the late-flowering phenotype of *Arabidopsis*.

### *MiCOL9A* and *MiCOL9B* play positive roles in combating abiotic stress

**Figure 9:**
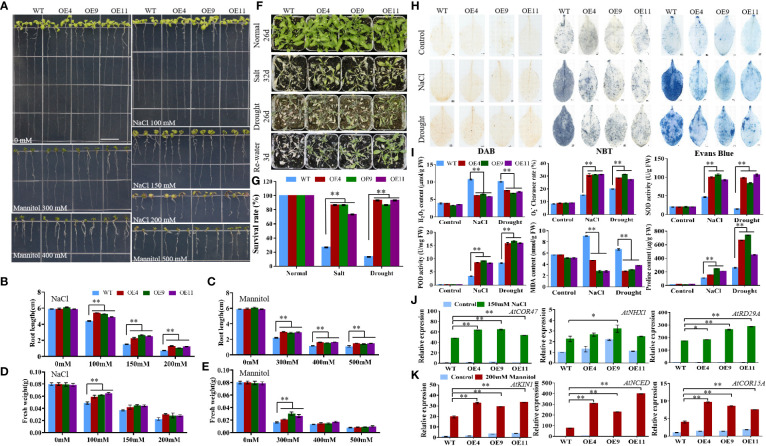
Analysis of *MiCOL9A* transgenic lines and WT under different stress treatments. **(A)** Phenotype. The bars represent 1.0 cm. **(B, C)** Root length and **(D, E)** fresh weight under NaCl and mannitol conditions. **(F)** Phenotypes. Normal, control; salt, 300 mM NaCl; drought, withholding of water. **(G)** Survival rate. **(H)** DAB, NBT and Evans Blue staining. **(I)** H_2_O_2_ content, ${\text{O}}_{2}^{-}$ clearance rate, SOD activity, POD activity, MDA content and proline content. **(J, K)** Expression pattern of stress-related genes. Blue, before treatment; green, 150 mM NaCl **(J)**; red, 200 mM mannitol **(K)**. Significant differences were assessed at *p*< 0.05 (*) and *p*< 0.01 (**) levels by Student’s *t* tests.

**Figure 10:**
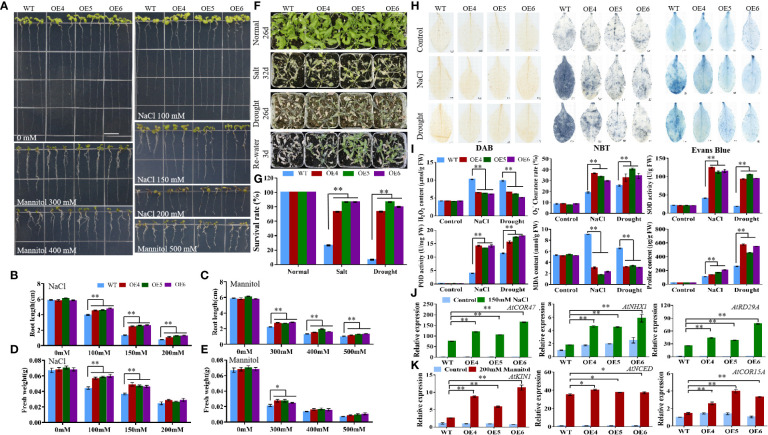
Analysis of *MiCOL9B* transgenic lines and WT under different stress treatments. **(A)** Phenotype. The bars represent 1.0 cm. **(B, C)** Root length and **(D, E)** fresh weight under NaCl and mannitol conditions. **(F)** Phenotypes. Normal, control; salt, 300 mM NaCl; drought, withholding of water. **(G)** Survival rate. **(H)** DAB, NBT and Evans Blue staining. **(I)** H_2_O_2_ content, ${\text{O}}_{2}^{-}$ clearance rate, SOD activity, POD activity, MDA content and proline content. **(J, K)** Expression pattern of stress-related genes. Blue, before treatment; green, 150 mM NaCl **(J)**; red, 200 mM mannitol **(K)**. Significant differences were assessed at *p*< 0.05 (*) and *p*< 0.01 (**) levels by Student’s *t* tests.

The T3 generation of *MiCOL9A* and *MiCOL9B* was selected to analyze the roles of these genes in combating abiotic stress. Three-day-old seedlings of transgenic and WT lines were transformed onto NaCl and mannitol stress media, and the primary root length and fresh weight were measured after 7 days of growth. There were no significant differences between untreated WT and transgenic plants. Under different stress treatments, the root length and fresh weight of the transgenic lines were always greater than those of the WT, and with increasing stress level, the difference between the transgenic lines and the WT gradually decreased (**Figures 9A-E**, **10A-E**).

To assess the stress response of the *MiCOL9A* and *MiCOL9B* genes, 7-day-old WT and transgenic lines were transplanted into square pots. After two weeks of growth, the plants were treated with 300 mM NaCl solution every 2 days for salt treatment or withheld water for drought treatment (**Figures 9F**, **10F**). Six days later, the survival rate of plants under salt stress was measured. The results showed that the survival rate of WT plants was approximately 25%, but the survival rates of both *MiCOL9A* and *MiCOL9B* transgenic lines were above 70% under salt stress. For drought treatment, the survival rate was measured after rewatering for 3 days. The results showed that the survival rate of WT plants was approximately 10%, but the survival rates of *MiCOL9A* and *MiCOL9B* transgenic lines were above 85% and 70%, respectively, under drought stress (**Figures 9G**, **10G**).

To further determine the molecular mechanism of *MiCOL9A* and *MiCOL9B* transgenic plants in response to salt and drought stresses, three salt- and drought-related genes were selected for expression pattern analysis. Under salt stress, the expression levels of salt-related genes in transgenic lines were significantly higher than those in WT, and all lines were significantly higher than those before treatment (**Figure 9J**, **10J**). Similarly, the expression levels of drought-related genes in transgenic lines were significantly higher than those in WT under drought stress, and all lines were significantly higher than those before treatment (**Figures 9K**, **10K**). In summary, these results suggest that the *MiCOL9A* and *MiCOL9B* genes enhance the tolerance of *Arabidopsis* to salt and drought stress.

### Physiological stress indexes

To investigate the ability of *MiCOL9A* and *MiCOL9B* transgenic lines to scavenge ROS under salt and drought conditions, we performed DAB and NBT staining of leaves of WT and transgenic plants. The results showed that the degree of stained leaves of transgenic lines under salt and drought treatment was slightly higher than that of other lines under normal conditions but was significantly lower than that of WT under stress (**Figures 9H**, **10H**). The H_2_O_2_ content and ${\text{O}}_{2}^{-}$ clearance rate results showed that the H_2_O_2_ content of the WT lines was significantly higher than that of the OE lines and that the ${\text{O}}_{2}^{-}$ clearance rate was significantly lower than that of the OE lines (**Figures 9I**, **10I**). Both the *MiCOL9A* and *MiCOL9B* transgenic lines were almost the same.

SOD and POD play a key role in ROS clearance and mainly remove ${\text{O}}_{2}^{-}$ and H_2_O_2_. We further measured the SOD and POD activities of WT and transgenic lines. According to the results, after stress treatment, the SOD and POD activities of the OE lines were significantly higher than those of the WT (**Figures 9I**, **10I**). These results indicate that *MiCOL9A* and *MiCOL9B* can improve the activity of SOD and POD under salt and drought conditions, thus reducing the accumulation of ROS in plants.

To assess the cell death and degree of membrane lipid peroxidation of WT and transgenic lines, we performed Evans Blue staining and measured MDA and proline content. As shown in **Figure 9H** and **Figure 10H**, the degree of stained leaves of OE lines under salt and drought treatment was significantly lower than that of WT under stress. The results showed that the MDA content was significantly higher in WT than OE lines under salt and drought conditions, but the proline content of WT lines was significantly lower than OE lines (**Figures 9I**, **10I**). This indicated that the *MiCOL9A* and *MiCOL9B* transgenic lines have stronger tolerance than WT to salt and drought stress conditions.

### MiCOL9A and MiCOL9B interacted with proteins

The Y2H Gold yeast cells contained *BD-MiCOL9A-ΔMR*, *BD-MiCOL9B-ΔMR* or BD-T7 and Y187 yeast cells contained candidate prey were mixed respectively, and coated onto DDO and QDO/X/A medium. Three days later, all combinations can grow on DDO medium, indicated that all mixed BD and AD formed binding. The BD-T7 and candidate prey mixture cannot grow on QDO/X/A medium, indicated that the candidate prey protein have no transcriptional activation activity. However, the mixture of *BD-MiCOL9A-ΔMR* and *BD-MiCOL9B-ΔMR* with candidate prey all grow normally and turned blue on QDO/X/A medium, indicated that *BD-MiCOL9A-ΔMR* and *BD-MiCOL9B-ΔMR* interacted with these proteins.

**Figure 11:**
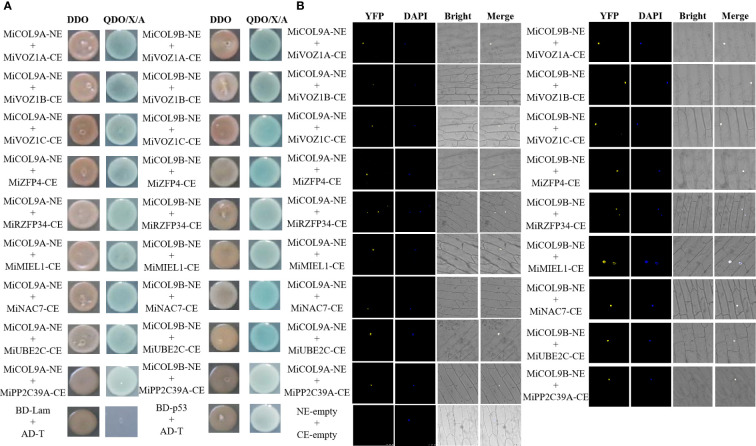
Identification of MiCOL9A and MiCOL9B interacting proteins. **(A)** Point-to-point verification. pGBKT7-Lam (BD-Lam) and pGADT7-T (AD-T) were used as negative controls, and pGBKT7-53 (BD-p53) and pGADT7-T (AD-T) served as positive controls. **(B)** BiFC assays. pSPYCE-empty and pSPYNE-empty were used as negative controls. Bars=100 μm.

According to the expression pattern analysis, both the *MiCOL9A* and *MiCOL9B* genes may be related to flowering and stress. To further investigate the function of *MiCOL9A* and *MiCOL9B*, we constructed MiCOL9A-NE and MiCOL9B-NE vectors and selected three flowering-related genes and six stress-related genes from the mango genome to construct the pSPYCE vector for the BiFC assay (**Figure 11**). The results demonstrate that MiCOL9A and MiCOL9B interact with these proteins in vivo.

## Discussion

*CO* and *COL* genes have been verified to play key roles in mediating light signaling in plant development (Suárez-López et al., 2001; Valverde et al., 2004). Since the first *CO* gene was isolated from *Arabidopsis* (Putterill et al., 1995), *COL* homologs have been identified from numerous plants (Griffiths et al., 2003; Cai et al., 2017). However, an investigation of the *CO* gene family in mango has yet to be conducted. In this study, we sequenced the whole genome of ‘JinHuang’ mango (unpublished), and 31 *COL* homologous genes were identified. The results of bioinformatic analysis showed that these 31 *MiCOL* gene family members, including five different structure types, were divided into three distinct clades (**Figure 1**) and unevenly distributed on 14 chromosomes (out of 20 in total) (**Figure 4A**). In contrast, *MiCOL4A*, *MiCOL4B* and *MiCOL11* contained only one BBX domain, consistent with the structural characteristics of the BBX gene family in *Arabidopsis* (Khanna et al., 2009), and a portion of *MiCOL*s in clade II contained only one CCT domain, consistent with the structural characteristics of the CCT motif family (CMF) in *Arabidopsis* (Griffiths et al., 2003; Khanna et al., 2009; Farreí and Liu, 2013). However, analysis of the sequences of these genes *via* NCBI Blast and phylogenetic trees revealed that these *MiCOL* genes were highly similar to other *CO* homologous genes and clustered with *MiCOL*s and *AtCOL*s.

A gene structure analysis showed that the *MiCO* genes in the same group have similar exons and introns, indicating a close relationship between evolution and gene structure. In this study, 31 *MiCOL*s were identified and contained ten motifs. Motif 2 was present in all genes except those in clade IV; motifs 3 and 4 existed simultaneously only in clade III; and motif 1 was absent only from *MiCOL4A*, *MiCOL4B* and *MiCOL11*. These findings suggest that motifs 2, 1 and 3/4 encode the B-box, CCT and DZF domains, respectively. According to our synteny analysis, the mango genome is collinear with the grape and *Populus trichocarpa* genomes and identified many orthologous gene pairs with the ‘Alfonso’ and ‘Tommy Atkins’ genomes, which might indicate that these genes have maintained high homology in the process of evolution.

According to a previous experiment, CO proteins are not only closely related to the effect of photoperiod on plant flowering but also involved in a variety of biological processes, such as stress responses and plant hormone signal transduction (Suárez-López et al., 2001; Song et al., 2008; Min et al., 2015). In this study, we analyzed cis-elements in the 2000 bp promoter regions of *MiCOL* genes. These genes mainly included light, circadian control, hormone and stress-responsive elements and were similar to genes in the *NtCOL* gene family (Zhang et al., 2021), *PaCOL* gene family (Khatun et al., 2021) and *PtCOL* gene family (Li et al., 2020a). The results imply that *MiCOL* genes might be involved in light, circadian control, and hormone and stress response pathways in mango.

*CO* is a light response gene and is widely expressed in different plant tissues, especially in the leaves (Suárez-López et al., 2001; Tan et al., 2016; Wu et al., 2017; Li et al., 2018). In this study, we analyzed the relative expression levels of *MiCOL*s in different tissues and developmental periods of mango *via* qRT–PCR. The results showed that most of the *MiCOL* genes were mainly expressed in leaves and had high expression levels during the floral induction and differentiation periods. Similarly, 9 *PtCOL* genes are predominantly expressed in leaves (Li et al., 2020), 5 *PaCOL* genes are preferentially expressed in stems (Khatun et al., 2021), and *BnaCO. A10* and *BnaCO. C9* transcripts accumulate to high levels in cauline leaves but to very low levels in flowers (Jin et al., 2021). In our previous study, *MiCO*, *MiCOL1A*, *MiCOL16A* and *MiCOL16B* all showed similar expression patterns (Liu et al., 2020; Guo et al., 2022; Liu et al., 2022). In contrast, four genes had high expression in flowers, similar to the *VviCOL* family genes, which are highly expressed in flower buds, suggesting that these genes may be involved in flowering induction (Wang et al., 2019).

In a previous study, most *CO*/*COL* genes, including the *AtCOL9*, *VviCOL9a*, *VviCOL9b* and *OsCOL9* genes, were reported to be located in the nucleus and to exert transcriptional activation activity through the middle regions between their domains (Cheng and Wang, 2005; Hao et al., 2016; Wang et al., 2019). Among these genes, *AtCOL9* has been confirmed to be located in the nucleus, and *VviCOL9a*, *VviCOL9b* and *OsCOL9* have all been confirmed to have transcriptional activation activity (Cheng and Wang, 2005; Hao et al., 2016; Wang et al., 2019). In this experiment, the *MiCOL9A* and *MiCOL9B* genes were confirmed to be located in the nucleus and display transcriptional activation activity through the MR domain, similar to the findings of *MiCOL1A*, *MiCOL1B*, *MiCOL16A* and *MiCOL16B* in previous research (Guo et al., 2022; Liu et al., 2022).

*CO* is the key gene of the photoperiodic pathway and promotes flowering in *Arabidopsis* (Putterill et al., 1995). However, overexpression of the *AtCOL4* and *AtCOL9* genes delays flowering time in transgenic *Arabidopsis* under LD conditions (Cheng and Wang, 2005; Min et al., 2015). Similarly, the *OsCOL9* gene inhibits flowering in rice under both LD and short-day (SD) conditions (Hao et al., 2016). In a previous study, the *MiCO*, *MiCOL1* and *MiCOL16* genes all inhibited flowering under LD and SD conditions (Guo et al., 2022; Liu et al., 2022). In this study, the flowering time of *MiCOL9A*- and *MiCOL9B*-transgenic lines was later than that of WT and pBI121 lines under LD conditions, and the numbers of rosette leaves in the transgenic lines were greater than those in the WT and pBI121 lines. These results indicate that the *MiCOL9A* and *MiCOL9B* genes in mango might suppress flowering.

During growth and development processes, plants are frequently threatened by various abiotic factors, such as heat, cold, drought, and salt (Peng et al., 2012). *CO*/*COL* genes have been well studied in the context of the regulation of flowering, but their roles in abiotic stress responses are unclear. To date, some studies have implied that *CO*/*COL* genes are related to abiotic stress responses. In *Arabidopsis*, *AtCOL4* expression is induced by salt and osmotic stress (Min et al., 2015). The *Ghd7* gene, a homolog of the *CO*-*like* gene, has been reported to regulate stress tolerance in rice (Liu et al., 2016). In the *PaCOL* gene family, the expression of 3 genes is upregulated significantly after heat, cold and drought stresses, and that of the *PaCOL2* gene is sharply elevated under salt conditions (Khatun et al., 2021). In this experiment, the expression levels of six genes were altered significantly after salt treatment, and seven genes were upregulated after drought treatment.

When plants are in adverse living environments, reactive oxygen species (ROS) accumulate and cause oxidative damage to cell components (Liu et al., 2010; Zhang et al., 2011b). H_2_O_2_ and ${\text{O}}_{2}^{-}$ are two major ROS and can be removed by SOD and POD, respectively, thereby increasing plant adaptability (Hossain et al., 2015). In addition, the product of membrane lipid peroxidation malondialdehyde (MDA) and plant cytoplasmic osmoregulation factor proline are also the main indicators of plant stress resistance (Rejeb et al., 2014; Tu et al., 2016). In this experiment, *MiCOL9A* and *MiCOL9B* transgenic plants showed stronger ROS removal ability than WT plants under both salt and drought stress conditions. The MDA content of OE lines accumulated significantly lower than WT, and the proline content was significantly higher than WT, indicating that the transgenic plants had better resistance to stresses. This is consistent with the results of the root length and survival rate experiments of the transgenic lines under salt and drought conditions. In addition, the expression levels of salt- and drought-related genes in the transgenic lines were all significantly higher than those in the WT under stress treatment, further proving that overexpression of the *MiCOL9A* and *MiCOL9B* genes can improve the resistance of *Arabidopsis* to salt and drought stresses. The results are also similar to those of *MiCOL1A*, *MiCOL1B*, *MiCOL16A* and *MiCOL16B* in previous research (Guo et al., 2022; Liu et al., 2022).

**Figure 12:**
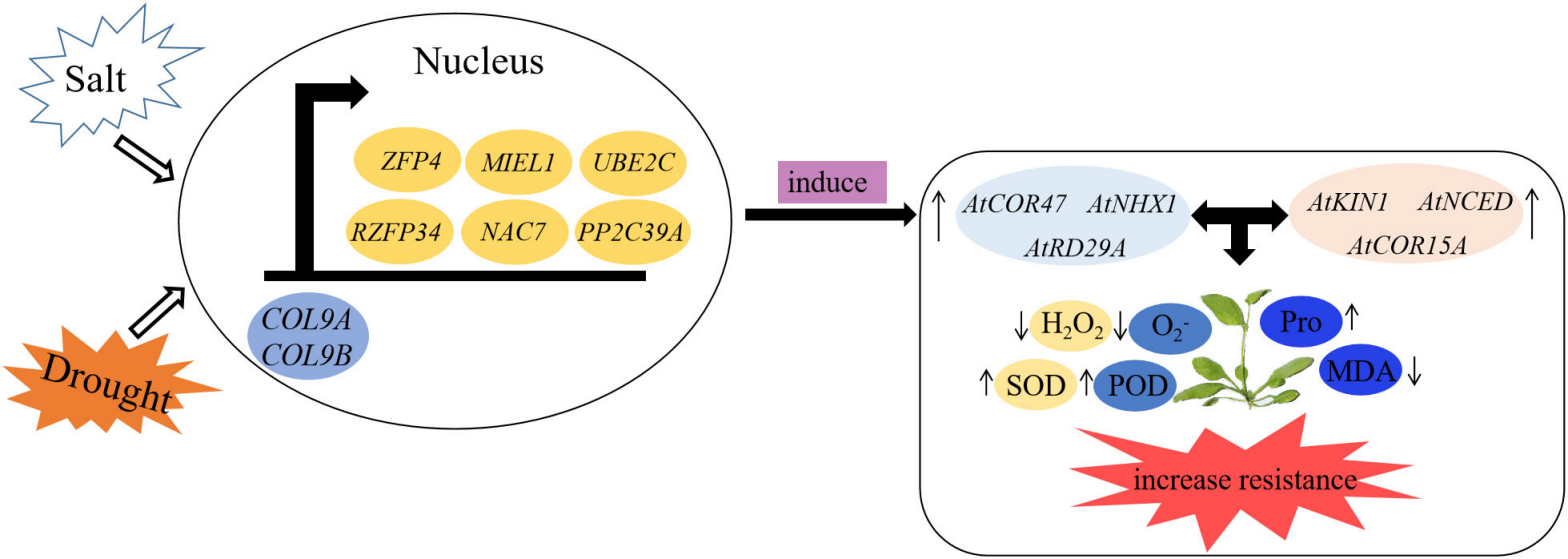
A model of *MiCOL9* in response to stresses in *Arabidopsis*. Under salt or drought condition, MiCOL9 interact with stress-relate proteins in nucleus, induced stress endogenous genes and then influenced the changes of relevant physiological indexes. Finally, increased the plants resistance to stresses.

CO has been confirmed to not only control flowering but also regulate plant resistance to abiotic stress. VOZs interact with CO and then activate FT to induce flowering (Valverde et al., 2004; Kumar et al., 2018), and *GhCOL* genes play diverse roles under stress (Qin et al., 2018). In this study, we screened three flowering-related proteins and six stress-related proteins in the mango genome for point-to-point and BiFC analysis, and both MiCOL9A and MiCOL9B interacted with these proteins. The model of plant responses to salt and drought stress showed that when plants are exposed to salt or drought stress, COL9 interacts with stress-related proteins in the nucleus to induce the expression of salt- or drought-related genes. Then, the SOD, POD and proline contents increased to remove peroxide and protect cells from damage. This further indicated that the MiCO gene may be involved in the regulation of plant flowering and stress resistance (**Figure 12**).

## Conclusion

In this study, we identified 31 *MiCOL* genes, including five structure types, which were divided into three clades. Bioinformatic analysis indicated that all genes were conserved and highly similar to *AtCOL*s in *Arabidopsis*. Expression pattern analysis showed that most members were mainly expressed in leaves and during the floral induction and differentiation periods. Functional analysis of *MiCOL9A* and *MiCOL9B* suggested that these two genes are located in the nucleus and have transcriptional activation activity, and overexpression of the *MiCOL9A* and *MiCOL9B* genes inhibited flowering in Arabidopsis under LD conditions. The expression levels of the *MiCOL9A* and *MiCOL9B* genes were affected by stress, and further work showed that the *MiCOL9A* and *MiCOL9B* genes improved the survival rate and ROS clearance ability by inducing stress-related gene expression, thereby increasing the resistance of *Arabidopsis* plants to salt and drought stress. Our results are helpful to better understand the function of *MiCOL* genes and further reveal the molecular mechanism by which the *MiCOL9A* and *MiCOL9B* genes improve plant stress resistance, which provides a theoretical basis for the genetic and breeding work of mango.

## Data availability statement

The original contributions presented in the study are included in the article/**Supplementary Material**. Further inquiries can be directed to the corresponding author.

## Author contributions

XH and CL conceived and designed the experiments. YL, RL, and ML performed this experiment. CL, HY, and YG provided technical assistance. SC, TL, and XM collected and analyzed the data. YL wrote the manuscript. CL and XH revised the manuscript. All authors contributed to the article and approved the submitted version.

## Funding

This research was supported by Science and Technology Major Projects of Guangxi (GXKJ- AA22068098-2), Innovation Team of Guangxi Mango Industry Project (nycytxgxcxtd-2021-06-02), The six one’ special action of “strengthening agriculture and enriching people” by science and technology Vanguard (Guangxi Agricultural Science and technology League 202204), Innovation Project of Guangxi Graduate Education (YCBZ2022024).

## Conflict of interest

The authors declare that the research was conducted in the absence of any commercial or financial relationships that could be construed as a potential conflict of interest.

## Publisher’s note

All claims expressed in this article are solely those of the authors and do not necessarily represent those of their affiliated organizations, or those of the publisher, the editors and the reviewers. Any product that may be evaluated in this article, or claim that may be made by its manufacturer, is not guaranteed or endorsed by the publisher.
