# Supplementary material for: Genome-wide identification of the mango CONSTANS (CO) family and functional analysis of two MiCOL9 genes in transgenic Arabidopsis
Source: Front Plant Sci. 2022 Oct 17;13:1028987. doi: 10.3389/fpls.2022.1028987 (PMC9618732; doi:10.3389/fpls.2022.1028987)
Supplement: Supplementary file 3 [file DataSheet_1.docx]

**Supplementary data**


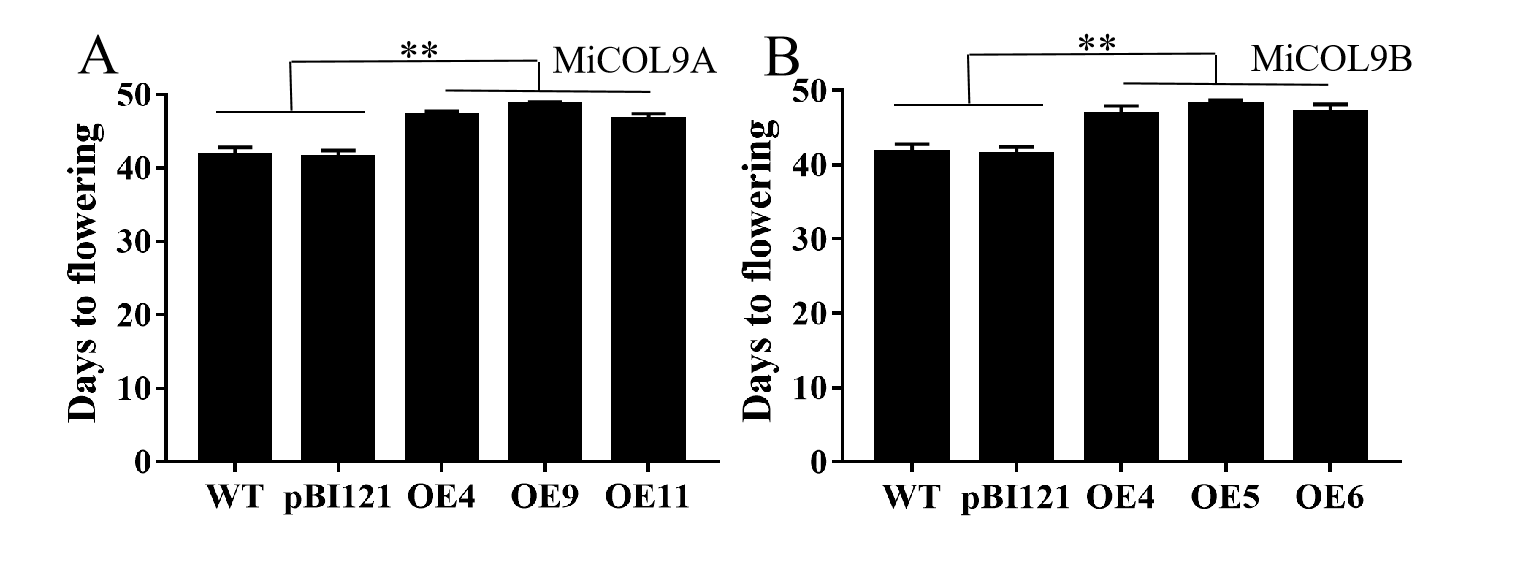


**Supplementary Figure 1 |** Ectopic expression of *MiCOL9A* and *MiCOL9B* delays flowering under short-day (SD) conditions. **(A-B)** Flowering times of *MiCOL9A* **(A)** and *MiCOL9B* **(B)** transgenic *Arabidopsis* plants under SD conditions.
